# Transcatheter Aortic Valve-in-Surgical Aortic Valve (TAV-in-SAV) for Structural Valve Degeneration of Post-Surgical Aortic Valve Replacement (SAVR) With the 3D-Nicks Procedure: A Case Report

**DOI:** 10.7759/cureus.115943

**Published:** 2026-09-08

**Authors:** Tomoaki Masuda, Atsuo Mori, Tadashi Omoto, Daisuke Itakura, Motoki Fukutomi

**Affiliations:** 1 Cardiovascular Surgery, Kawasaki Municipal Hospital, Kawasaki, JPN; 2 Cardiovascular Surgery, Showa University, Tokyo, JPN; 3 Cardiology, Kawasaki Saiwai Hospital, Kawasaki, JPN

**Keywords:** aortic valve surgery, bioprosthetic aortic valve, deterioration, nick's root enlargement, patient prosthetic mismatch(ppm), redo surgical aortic valve replacement, small aortic annulus, structural valve deterioration, trans catheter aortic valve implantation (tavi), valve-in-valve

## Abstract

Transcatheter aortic valve implantation (TAVI) for structural valve deterioration (SVD) following surgical aortic valve replacement (SAVR) is an established alternative to redo surgery (transcatheter aortic valve-in-surgical aortic valve, or TAV-in-SAV). However, in patients with a small aortic annulus or small sinus of Valsalva (SOV), the risk of coronary artery obstruction or severe prosthesis-patient mismatch (PPM) remains a major constraint. To address this, we previously introduced the "3D-Nicks" procedure, using a preoperatively prepared three-dimensional ellipsoidal patch to achieve circumferential aortic root enlargement. We report a 14-year successful clinical course of TAV-in-SAV following SAVR with the 3D-Nicks procedure. A 61-year-old woman with severe aortic valve stenosis (AS) and a narrow aortic root (annulus 19 mm, STJ 20 mm) underwent SAVR (CEP19^®︎^ mm, Edwards Lifesciences, Irvine, CA) combined with the 3D-Nicks technique. Around 14 years postoperatively, she developed severe SVD. Contrast CT demonstrated that 3D-Nicks had maintained ample root geometry (SOV 30.2-36.8 mm, STJ 31.8 mm, VTC > 5.0 mm, VTSTJ > 6.0 mm). TAV-in-SAV was successfully performed using a 23 mm Evolut FX^®︎^ valve (Medtronic, Minneapolis, MN) via transfemoral access without coronary protection. Postoperative echocardiography showed excellent hemodynamics (mean PG 6 mmHg, EOAi 1.29 cm²/m²), which remained stable at the three-year follow-up (EOAi 1.16 cm²/m²). This case highlights that the 3D-Nicks procedure may provide favorable three-dimensional aortic root dimensions that facilitate safe long-term TAV-in-SAV.

## Introduction

Structural valve deterioration (SVD) of bioprosthetic aortic valves after surgical aortic valve replacement (SAVR) leads to valvular dysfunction, limiting long-term valve durability. As patient life expectancy increases, initial SAVR strategies must incorporate lifetime management planning for potential secondary interventions. Transcatheter aortic valve implantation (TAVI) within a failed surgical bioprosthesis, termed transcatheter aortic valve-in-surgical aortic valve (TAV-in-SAV), has emerged as a less invasive alternative to redo surgery. However, performing TAV-in-SAV safely in patients with a small aortic root is challenging due to two interconnected risks: prosthesis-patient mismatch (PPM) and coronary artery obstruction.

PPM occurs when the effective orifice area (EOA) of the implanted valve is too small relative to the patient’s body surface area (BSA), increasing long-term mortality [1]. Because placing a transcatheter heart valve (THV) inside a pre-existing surgical frame naturally reduces the internal diameter, TAV-in-SAV carries an inherently high risk of severe PPM. Conversely, coronary obstruction during TAV-in-SAV occurs when the newly expanding THV displaces the rigid surgical bioprosthetic leaflets outward. In a small root, these deflected leaflets can directly cover the coronary ostia or seal off the sinus of Valsalva (SOV), causing sinus sequestration and preventing coronary perfusion [2,3]. Anatomical safety margins are governed by the valve-to-coronary (VTC) distance and valve-to-sinotubular junction (VTSTJ) distance. Up to 34.8% of post-SAVR aortic valve stenosis (AS) patients are estimated to be at high risk for coronary obstruction during future TAV-in-SAV due to inadequate clearances [4].

In patients with a small aortic annulus and narrow root, surgeons face a critical clinical trade-off. To prevent PPM, aortic annular enlargement is often performed to accept a larger bioprosthesis. Paradoxically, placing a larger surgical frame into a narrow root positions the valve closer to the aortic wall, dramatically reducing baseline VTC and VTSTJ clearances and increasing the risk of fatal coronary obstruction during subsequent TAV-in-SAV [4].

Conventional aortic annular enlargement techniques, such as the standard Nicks procedure, utilize a flat, two-dimensional patch to widen the annulus, which often results in asymmetric, planar expansion without restoring natural sinus volume. Advanced techniques like the Y-incision allow for extensive enlargement but carry greater surgical complexity [5]. In contrast, the modified "3D-Nicks procedure" utilizes a pre-shaped, three-dimensional ellipsoidal patch. Rather than simply widening the annulus in a single plane, this technique has the potential to naturally reconstruct the volumetric bulging geometry of the SOV up to the sinotubular junction (STJ), restoring generous VTC and VTSTJ safety clearances while maintaining procedural simplicity.

Although initial root enlargement to facilitate future TAVI has generated considerable interest, a significant knowledge gap remains: real-world clinical evidence demonstrating successful, long-term TAV-in-SAV following prior annular/root enlargement is extremely limited. Here, we report a case of successful TAV-in-SAV performed 14 years after initial SAVR with the 3D-Nicks procedure, demonstrating how three-dimensional root reconstruction safely prevented coronary obstruction and provided optimal long-term hemodynamics.

## Case presentation

Initial SAVR procedure (age 61)

A 61-year-old woman presented with loss of consciousness, chest pain, and lower extremity edema. On physical examination, her height, weight, and BSA were 158.8cm, 47.6kg, and 1.41m^2^, a systolic ejection murmur was heard at the right upper sternal border. Echocardiography demonstrated severe AS (EF 61.4%, max PG 105.8 mmHg, mean PG 59.7 mmHg), and surgery was decided. Echocardiography also revealed a narrow aortic root morphology with an aortic annulus diameter of 19 mm, SOV diameter of 27 mm, SOV height of 13 mm, and STJ diameter of 20 mm (Figure 1). Therefore, SAVR with the 3D-Nicks procedure was planned.

**Figure 1:**
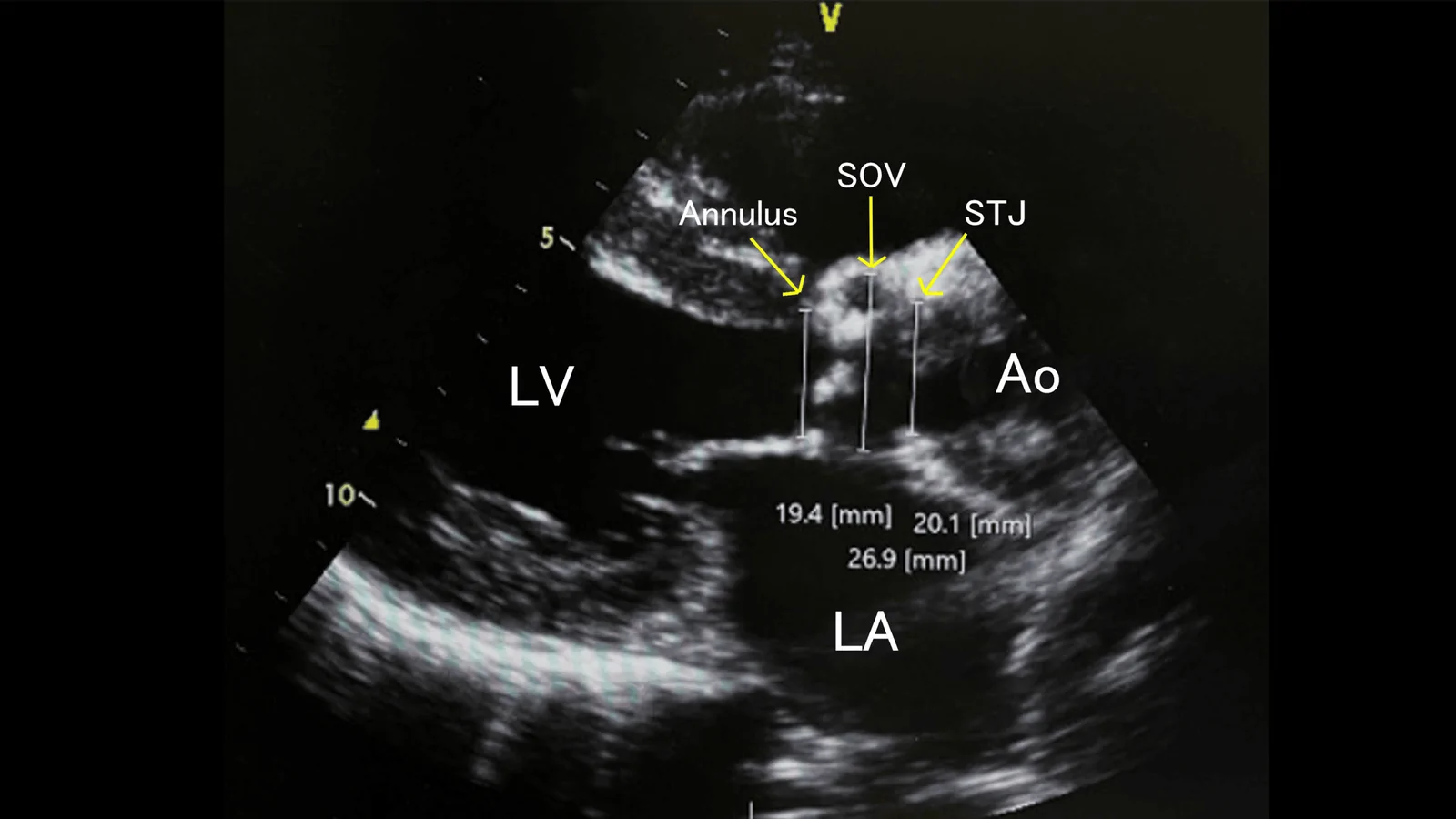
Preoperative echocardiography Preoperative echocardiographic measurements revealed a narrow aortic root morphology with an aortic annulus diameter of 19 mm, sinus of Valsalva (SOV) diameter of 27 mm, and sinotubular junction (STJ) diameter of 20 mm. LV, left ventricle; LA, left atrium; Ao, aorta.

Thoracotomy was performed via median sternotomy. Autologous pericardium was harvested in a spindle shape. Cardiopulmonary bypass was established using standard methods. Cardiac arrest was achieved by antegrade, retrograde, and selective cardioplegia administration. The aorta was obliquely incised. The aortic valve was markedly hardened, and fusion was observed at the R-N commissure. After aortic resection, valve sizing was attempted; however, even the smallest sizer (annular barrel type (19 mm diameter)) could not pass through the annulus, and the STJ was also narrow, making sizer passage extremely tight.

Given this patient's physique, it was thought that severe PPM could be avoided if even a PERIMOUNT Magna (CEP)^®︎^19mm (Edwards Lifesciences, Irvine, CA) valve could be inserted, and that annular dilation could be achieved using the Nicks procedure. On the other hand, in this case, the outer diameter of the stent posts for the CEP^®︎^19mm valve was approximately 20 mm, while the STJ height was lower than the stent posts and the STJ diameter was around 20 mm. There was a risk that the effective diameter would narrow further upon closure of the aortotomy, considering the suture margins--potentially leading to complications such as accidental suturing of the valve leaflets and distortion of the stent posts due to contact with the aortic wall. Therefore, to ensure a safe procedure, we determined that the supra-annular aortic root geometry needed to be enlarged in three dimensions, and we employed the 3D-Nicks technique.

We estimated that enlarging the diameter from 20 mm to approximately 30 mm would ensure safety. Calculating the difference in circumference (approx. 35 mm) plus the suture margins (approx. 5 mm) suggested a patch width of about 40 mm; we designed the patch accordingly (Figure 2A). (Strictly speaking, the patch is applied three-dimensionally and somewhat obliquely along the J-shaped aortotomy, but we considered that precise calculation was unnecessary given the margin of error inherent in suturing.)

**Figure 2:**
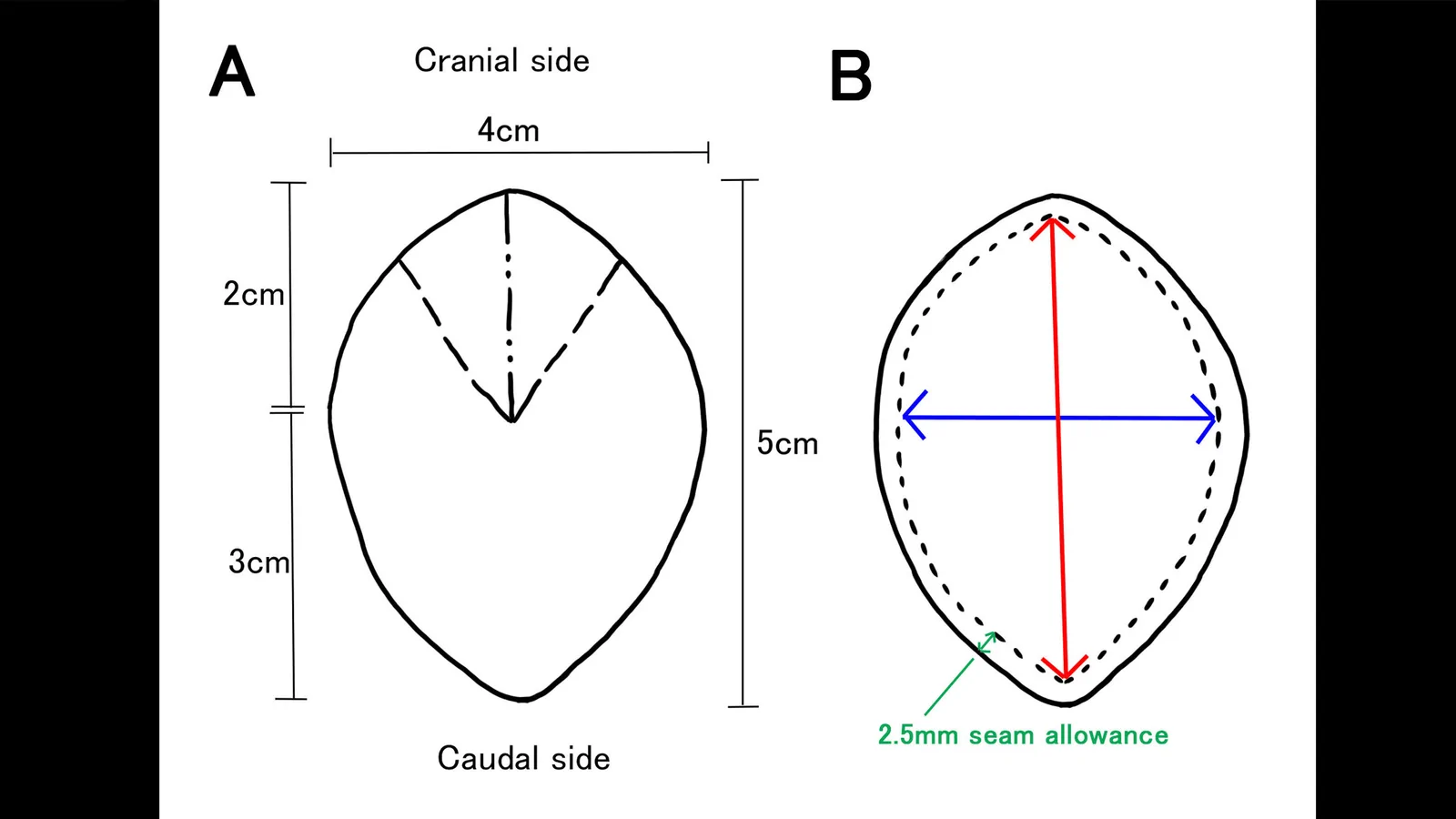
3D patch design in the 3D-Nicks procedure A: Patch design for this case. B: How to determine the size of 3D patches. Green line: Approximately 2.5 mm seam allowance. Blue line: Difference in circumference between the aortic diameter and the target aortic diameter. Red line: Desired height for patch expansion. Image credit: Created by the authors.

Following the Nicks procedure, an incision was made in the annulus from the non-coronary cusp (NCC), and an ellipsoidal three-dimensional patch made by layering autologous pericardium and Hemashield^®︎^ (Maquet Cardiovascular, Wayne, NJ) (Figure 3A-3B) was applied and enlarged with 4-0 Prolene^®︎^(Ethicon, Somerville, NJ) continuous sutures (Figure 3C). CEP^®︎^19mm valve was transplanted to the aortic annulus in the supra-annular position using horizontal mattress sutures with 2-0 Ethibond^®︎^(Ethicon, Somerville, NJ) stitches (Figure 3D-3E). The use of the 3D-Nicks technique secured adequate, safe suturing space throughout the procedure--from valve replacement to aortotomy closure--allowing the surgery to be performed without complications. In this operation, the cardiopulmonary bypass time was 186 minutes, and the aortic cross-clamp time was 127 minutes.

**Figure 3:**
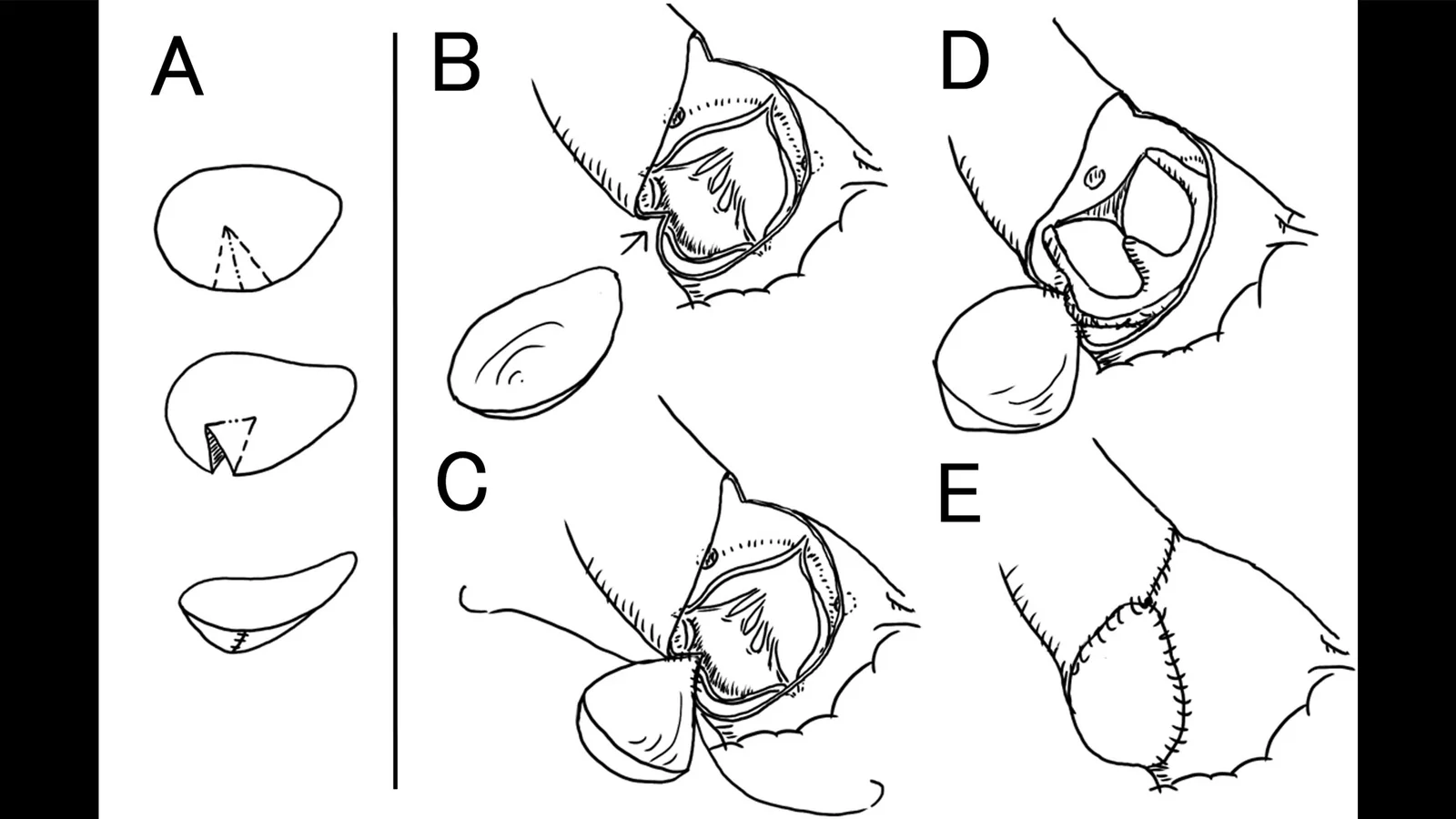
3D-Nicks procedure A: The 3D patch is created by suturing the autologous pericardium to the inside of a spindle-shaped Hemashield^®︎^ (Maquet Cardiovascular, Wayne, NJ, USA), folding it, and then sewing it to form a three-dimensional shape. B: The 3D patch is applied to the incision made in the direction of the non-coronary cusp. C: Before implanting the bioprosthetic valve, the 3D patch is applied to the subvalvular incision and partially sutured. D: A bioprosthetic valve is implanted. When placing sutures for valve implantation at the patch site, the sutures are passed from the outside of the patch. E: Tie the sutures coming up from both sides of the 3D patch and the suture coming up from the left to close the aortotomy. Image credit: Created by the authors.

The postoperative course was uneventful. The patient was weaned off the ventilator the day after surgery and moved to a general ward on the third postoperative day. Postoperative echocardiogram showed the effective orifice area index (EOAi) of 0.89, indicating low risk of PPM. The patient was discharged on the 33rd postoperative day after completion of the rehabilitation program.

SVD progression after SAVR (age 75)

Around 14 years after the operation at the age of 75 (height 149.0 cm, weight 42.0 kg, and BSA 1.30 m^2^), the patient developed exertional dyspnea, atrial fibrillation, and a systolic murmur on the right sternal border. Annual echocardiography showed that the valve function had been normal until then, but the mean pressure gradient had increased over the past eight months (mean PG from 15.8 mmHg to 43.4 mmHg). Valvular regurgitation was limited to trivial transvalvular leakage. She was diagnosed with progressive AS due to SVD. The patient was hospitalized and treated for heart failure, but due to a rapid drop in blood pressure and falls caused by syncope, surgery was decided upon. The patient also had diabetes and rheumatoid arthritis (using immunosuppressants and steroids), and a decline in activities of daily living (ADL) had been observed since the initial surgery. With a CSHA score of 3, NYHA class 3, Euro II score of 8.56%, and STS score of 8.15%, redo SAVR was considered high-risk, and TAV-in-SAV was recommended after heart team conference.

TAV-in-SAV Procedure and Outcomes

TAVI measurements were performed using contrast-enhanced CT (Table 1) (Figure 4). All pre-TAVI CT measurements were obtained using 3D multiplanar reconstruction (MPR) during the end-systolic phase (20-30% of the R-R interval) to reflect maximum root dimensions and safety clearances. The true inner diameter (ID) of the bioprosthetic valve was 17.8 mm on CT measurement. Due to the 3D-Nicks procedure, the SOV and STJ were maintained at sufficient size. Since the SOV diameter was 25 mm or greater, it was possible to deploy the Evolut FX®23 mm (Medtronic, Minneapolis, MN) for TAV-in-SAV in a case with a true ID of 17.8 mm bioprosthetic valve (Figure 4A). There was sufficient distance to the left and right coronary arteries (4 mm or more), and the risk of coronary artery occlusion was considered low (Figure 4B). The STJ was sufficiently enlarged by the 3D patch, and the surgical bioprosthetic valve was positioned almost centrally (Figure 4C). The VTSTJ distance was at least 3.5 mm in all directions, and the risk of coronary artery obstruction was considered low (Figure 4D). CT volume-rendered images of the aortic root also show that the surgical bioprosthetic valve was positioned vertically near the center of the aortic root, which had been adequately enlarged using a 3D patch (Figure 4E); consequently, the risk of coronary artery obstruction was considered low. Based on measurements, it was determined that the Evolut FX^®^23 mm--a self-expanding, supra-annular position THV--could be implanted; this device was compatible with TAV-in-SAV for a CEP^®^19 mm valve and was expected to achieve an effective orifice area (EOA) that avoids PPM. There were no issues with the access artery for the transfemoral artery approach. Without the need for coronary artery protection, TAV-in-SAV was successfully performed using a 23 mm Evolut FX^®︎^ via a right femoral artery approach. The THV was deployed perpendicularly to the SAV at an appropriate height (approximately 3 mm below the valve) in the perpendicular view of the SAV without the need for recapture. After placement, there was no atrioventricular block, and the final contrast showed that there was sufficient space from the outside of the SAV to the SOV; there were no problems with coronary artery blood flow, and there was no paravalvular regurgitation (Figure 5). Furthermore, coronary re-engagement was considered feasible due to the preserved VTC/VTSTJ clearance and appropriate commissural alignment during Evolut FX^®^deployment.

**Table 1:** Longitudinal echocardiographic data and pre-TAV-in-SAV CT measurements SAVR, surgical aortic valve replacement; TAV-in-SAV, transcatheter aortic valve-in-surgical aortic valve; LVEF, left ventricular ejection fraction; PG, pressure gradient; EOA, effective orifice area; EOAi, effective orifice area index; AR, aortic valve regurgitation; ID, Inner diameter; SOV, sinus of Valsalva; STJ, sinotubular junction; VTC, valve-to-coronary; LM, left main trunk; RCA, right coronary artery; VTSTJ, valve-to- sinotubular junction.

Parameter	Pre-SAVR (Age 61)	Post-SAVR Baseline	Pre-TAV-in-SAV (Age 75)	Post-TAV-in-SAV (Immediate)	Post-TAV-in-SAV (3-Year Follow-up)
Echocardiography					
LVEF (%)	61.4	69.9	78.0	68.0	76.5
Max PG (mmHg)	105.8	29.2	67.2	11.0	10.2
Mean PG (mmHg)	59.7	17.5	43.4	6.0	5.0
EOA (cm²)		1.25		1.68	1.47
EOAi (cm²/m²)		0.89		1.29	1.16
AR	No	trivial	trivial	trivial	trivial
Aortic root Mmeasurements	Echocardiography		CT		
Annulus diameter (mm)	19		17.8 (true ID)		
SOV diameter / width (mm)	27		L: 36.8 / R: 32.0 / N: 30.2		
SOV height (mm)	13		L: 23.2 / R: 38.8 / N: 38.0		
STJ diameter (mm)	20		31.8		
VTC distance LM / RCA (mm)			LM: 6.0 / RCA: 5.2		
VTSTJ distance LM / RCA (mm)			LM: 8.4 / RCA: 6.1		

**Figure 4:**
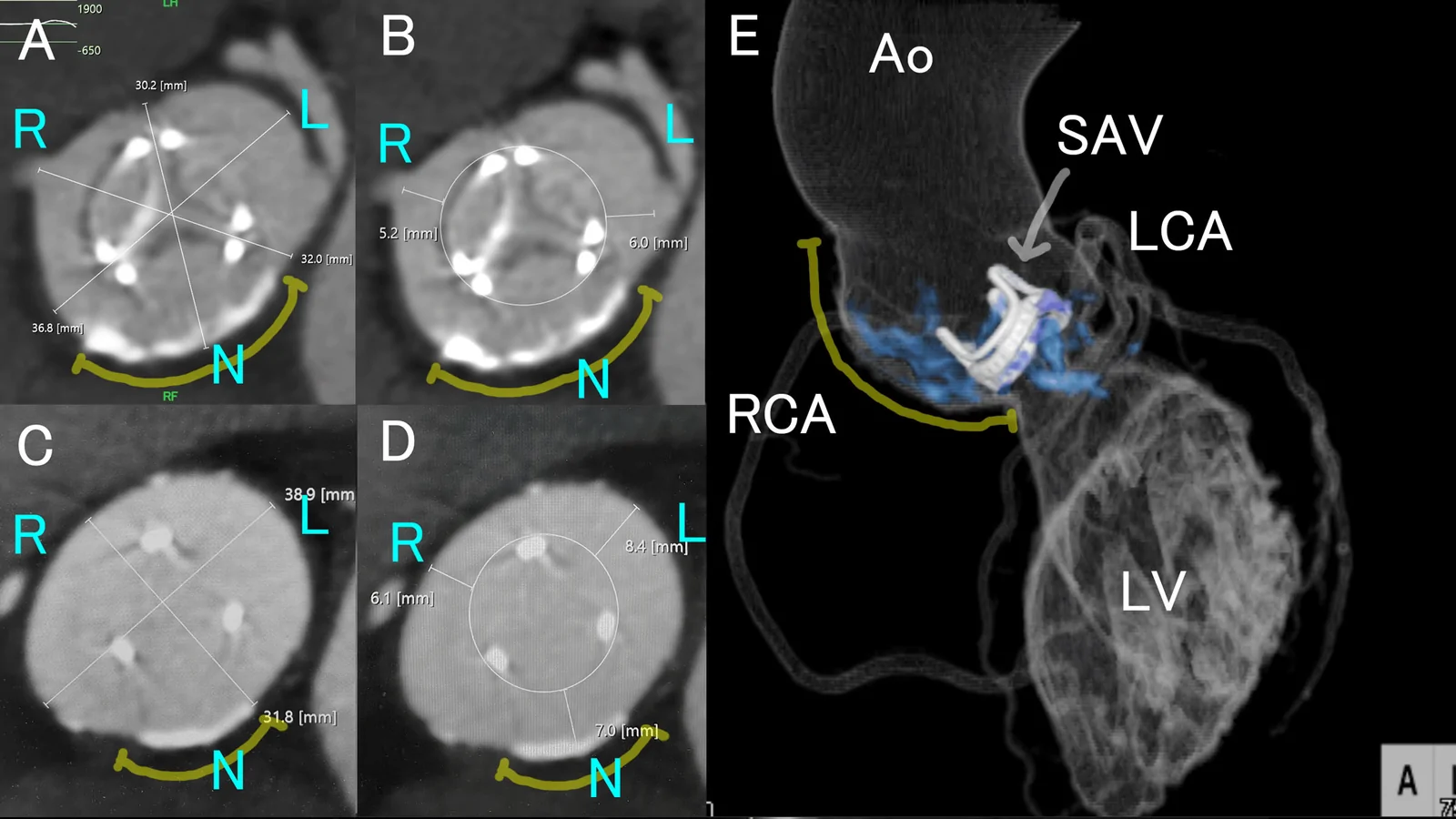
Preoperative measurement of TAV-in-SAV (transcatheter aortic valve implantation in surgical aortic valve) by contrast-enhanced CT. Yellow line: The area where the 3D patch is applied A: Sinus of Valsalva (SOV) width B: Valve-to-coronary (VTC) distance C: Sinotubular junction (STJ diameter) D: Valve-to-sinotubular junction (VTSTJ) distance E: Aortic root CT volume rendering R, right coronary cusp side; L, left coronary cusp side; N, non-coronary cusp side; Ao, aorta; SAV, surgical aortic valve; RCA, right coronary artery; LCA, left coronary artery; LV, left ventricle.

**Figure 5:**
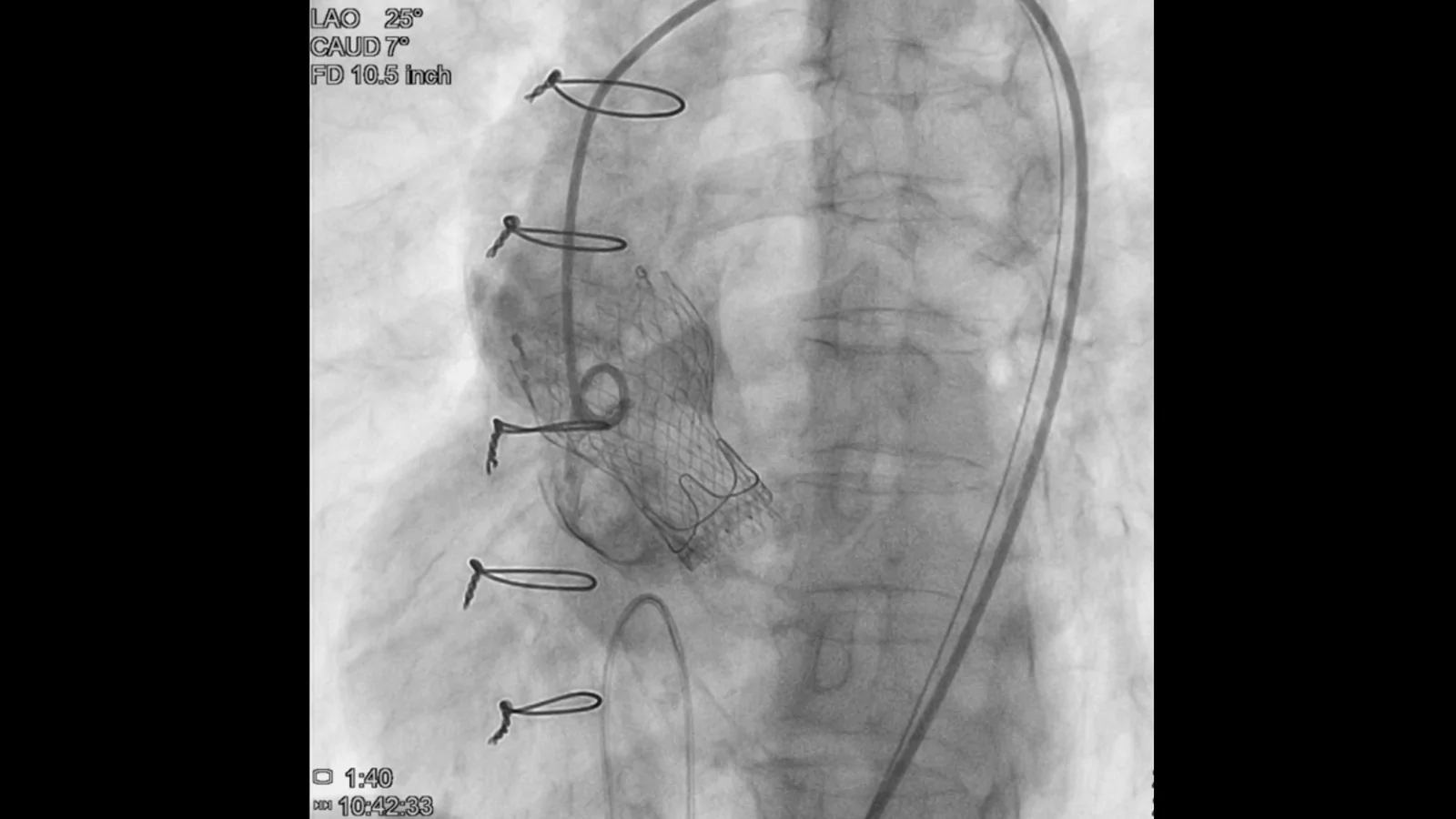
Intraoperative fluoroscopic image of transcatheter aortic valve implantation in surgical aortic valve (TAVI-in-SAV): Aortic root angiography following Evolut FX® implantation. For structural valve deterioration following surgical aortic valve replacement (CEP^®^ 19mm) + 3D-Nicks procedure, TAV-in-SAV (Evolut FX® 23mm) was performed without any problems.

Postoperatively, echocardiography showed good valve function with an EF of 68%, mean PG of 6 mmHg, EOA of 1.68 cm^2^, and EOAi of 1.29 cm^2^/m^2^.

Three years after the TAV-in-SAV procedure, the patient is doing well and continues to attend outpatient follow-ups. The patient has maintained NYHA class I status, and an echocardiogram performed three years postoperatively shows good valve function with an EOAi of 1.16 (Table 1).

## Discussion

Considering that the reported durability of surgical bioprosthetic valves is approximately 15 years [6], many patients who undergo bioprosthetic valve SAVR survive beyond the durability of the bioprosthetic valve and require redo SAVR. Recent advances in valve-in-valve TAVI have made minimally invasive TAV-in-SAV an attractive surgical option for SVD after SAVR [7].

However, unlike redo SAVR, TAVI has several important anatomical limitations; access and, especially, the morphology of the aortic root are crucial for performing the procedure safely. Furthermore, in TAV-in-SAV, assessment of the risk of coronary artery occlusion is also important. TAV-in-SAV has been reported to carry a significantly higher risk of coronary artery occlusion than conventional TAVI [8], due to the stent frame of the TAVI valve fixing the leaflets of the surgical bioprosthetic valve in an open position, resulting in direct coronary artery occlusion and sinus sequestration [2,3]. Although the risk of coronary artery occlusion is around 2-6%, the mortality rate is very high at approximately 40%, which limits the option of TAV-in-SAV in patients with small aortic root anatomy [9,10].

The criteria for evaluating the avoidance of coronary artery occlusion risk during TAVI-in-SAV include a VTC wider than 4 mm and a VTSTJ wider than 3.5 mm [9,11,12]. Furthermore, Kawamura et al. published a study that simulated the risk of coronary artery occlusion in future TAV-in-SAV based on the anatomical structure of the aortic root before surgery [4]. According to this report, the most important indicator of coronary artery occlusion was identified as "expected VTSTJ" (preoperative STJ diameter - predicted inner diameter of implanted valve). In other words, from the perspective of avoiding the risk of coronary artery occlusion, it is necessary to select a valve with a smaller inner diameter in the initial SAVR to ensure VTSTJ.

This presents a conflicting set of conditions in TAV-in-SAV using an intra-annular position THV, from the perspective of avoiding postoperative PPM. There are reports that if EOAi falls below 0.65 PPM after TAVI-in-SAV, the mortality rate is significantly increased [1]. To avoid PPM, procedures such as bioprosthetic valve fracture (BVF), which involves placing a larger THV by destroying the existing stent frame with a high-pressure balloon [13], and the use of the Inspiris Resilia®︎ bioprosthetic valve (Edwards Lifesciences, Irvine, CA), which has a structure that expands the stent frame during TAV-in-SAV, are used. However, the aforementioned expected VTSTJ ultimately decreases as the inner diameter of the implanted valve increases, and the risk of coronary artery occlusion increases. In the intra-annular position, THV requires achieving both a significant increase in the size of the valve annulus and a correspondingly significant enlargement of the supravalvular aortic root, which may increase the invasiveness of the initial SAVR. There are also reports of the BASILICA method [14], in which the bioprosthetic valve near the coronary artery ostium is cut with an electrically charged wire in patients at high risk of coronary artery occlusion, but it is unlikely to become a standard procedure.

On the other hand, supra-annular position THV such as Evolut FX^®^ can achieve a larger EOA than SAV. Therefore, as in this case, minimal annular dilation to avoid postoperative PPM and three-dimensional patch expansion of SOV that allows for safe placement of supra-annular position THV in the future is considered one of the minimally invasive surgical strategies.

Recently, there have been several reports of SAVR with annular dilation being performed to enable TAV-in-SAV in the future [15-17], and many of these use the Y-incision procedure, which allows for significant annular dilation [5]. On the other hand, there are few case reports of actual TAV-in-SAV being performed after SAVR with annular dilation, with the only available report being by Jelisejevas et al. [18]. A 47-year-old woman underwent SAVR (CEP^®^21mm) with annular dilation for infective endocarditis (IE). Nine years later, at age 56, she was diagnosed with SVD. CT measurements showed VTC-left main 3.5mm, VTC-right coronary artery 3.0mm, and VTSTJ-left main trunk 3.0mm. Therefore, TAV-in-SAV (Sapien XT®20 mm (Edwards Lifesciences, Irvine, CA)) was performed with left coronary artery protection. However, 10 years later, at age 66, she was diagnosed with SVD again, but VTSTJ-left main trunk was 1.5mm, making TAVI difficult. Therefore, considering her complications (chronic kidney disease, end-stage liver disease, history of cardiac surgery), although SAVR was also high-risk, she underwent a third surgery, SAVR (Inspiris^®^23mm). Although details such as the surgical procedure for annular dilation are unknown, this case report highlights the importance of avoiding the risk of coronary artery occlusion during the initial SAVR surgery in order to leave open the option of TAV-in-SAV in the future [18].

To contextualize our findings, we compared our case with the report by Jelisejevas et al. (Table 2). While Jelisejevas et al. required coronary protection during TAV-in-SAV due to tight VTC/VTSTJ clearances (~3.0 mm) and eventually required a high-risk third reoperation for late SVD [18], our 3D-Nicks technique provided robust, three-dimensional root enlargement that allowed straightforward Evolut FX deployment with optimal hemodynamics (mean PG 6 mmHg, EOAi 1.29 cm^2^/m^2^) and no coronary compromise.

**Table 2:** Comparison of TAV-in-SAV outcomes after prior aortic root enlargement between Jelisejevas et al. and the present case. SAVR, surgical aortic valve replacement; AS, aortic valve stenosis; SVD, structural valve deterioration; TAV-in-SAV, transcatheter aortic valve-in-surgical aortic valve; TAVI, transcatheter aortic valve implantation; VTC, valve-to-coronary; LM, left main trunk; RCA, right coronary artery; VTSTJ, valve-to-sinotubular junction; LCA, left coronary artery; EOAi, effective orifice area index; THV, transcatheter heart valve.

Clinical parameters	Jelisejevas et al. [18]	Present case (3D-Nicks)
Age/sex at initial SAVR	47/Female	61/female
Indication	Infective endocarditis	Severe AS
Surgical detail	Annular dilation	3D-Nicks procedure
Initial SAVR valve	CEP® 21mm	CEP® 19 mm
Time to SVD/TAV-in-SAV	9 years	14 years
Pre-TAVI CT clearances		
VTC distance LM	3.5 mm	6.0 mm
VTC distance RCA	3.0 mm	5.2 mm
VTSTJ distance LM	3.0mm	8.4 mm
TAV-in-SAV valve implanted	Sapien XT 20® mm	Evolut FX® 23 mm
Coronary protection required?	Yes (LCA protection)	No
Late outcome	Recurrent SVD at 10 years; redo-SAVR required due to tight VTSTJ (1.5 mm)	Stable at 3-year follow-up (EOAi 1.16 cm²/m², NYHA Class I)
Key clinical insight	Conventional root enlargement left borderline VTC/VTSTJ, leading to surgical redo upon 2nd SVD	3D-Nicks achieved circumferential root expansion, enabling safe supra-annular THV deployment

Aortic annulus enlargement procedures aim to enable the implantation of an artificial valve of the desired size when the native annulus is too small; among these, the Nicks procedure--which involves making an incision and enlarging the annulus on the NCC side, where an incision causes the least disruption--is the simplest and easiest procedure.

While the Nicks procedure achieves an annular enlargement of approximately one size, other methods allow for more extensive enlargement of the annulus (such as the Y-incision extending beneath the NCC) or enlargement that includes the left ventricular outflow tract (such as the Manouguian procedure, where the incision is extended to the anterior leaflet of the mitral valve); these various conventional methods primarily focus on enlarging the annulus and the sub-annular region. In contrast, the 3D-Nicks technique limits annular enlargement to that of the simplest Nicks procedure, focusing instead on enlarging the supra-annular structures, such as the SOV and the STJ.

The 3D-Nicks method has the following two features: (1) Because the patch is three-dimensional, it can be expanded while retaining its bulging SOV form, maintaining the distance between the bioprosthetic valve and the aortic wall (securing VTC). (2) In addition, by applying the patch to the J-shaped aortotomy, the patch can be expanded to include the STJ (securing VTSTJ).

The purpose of the aortic annulus expansion procedure is to insert a valve of the desired size by cutting into the annulus and enlarging the patch when the annulus is too small to fit an artificial valve of the desired size. Since the patch is usually flat, unlike the SOV form, which originally has a bulge, only the patch part is formed flat, and the distance between the biological valve and the aortic wall becomes closer, and it may not be expanded uniformly all around the circumference (Figure 6B).

**Figure 6:**
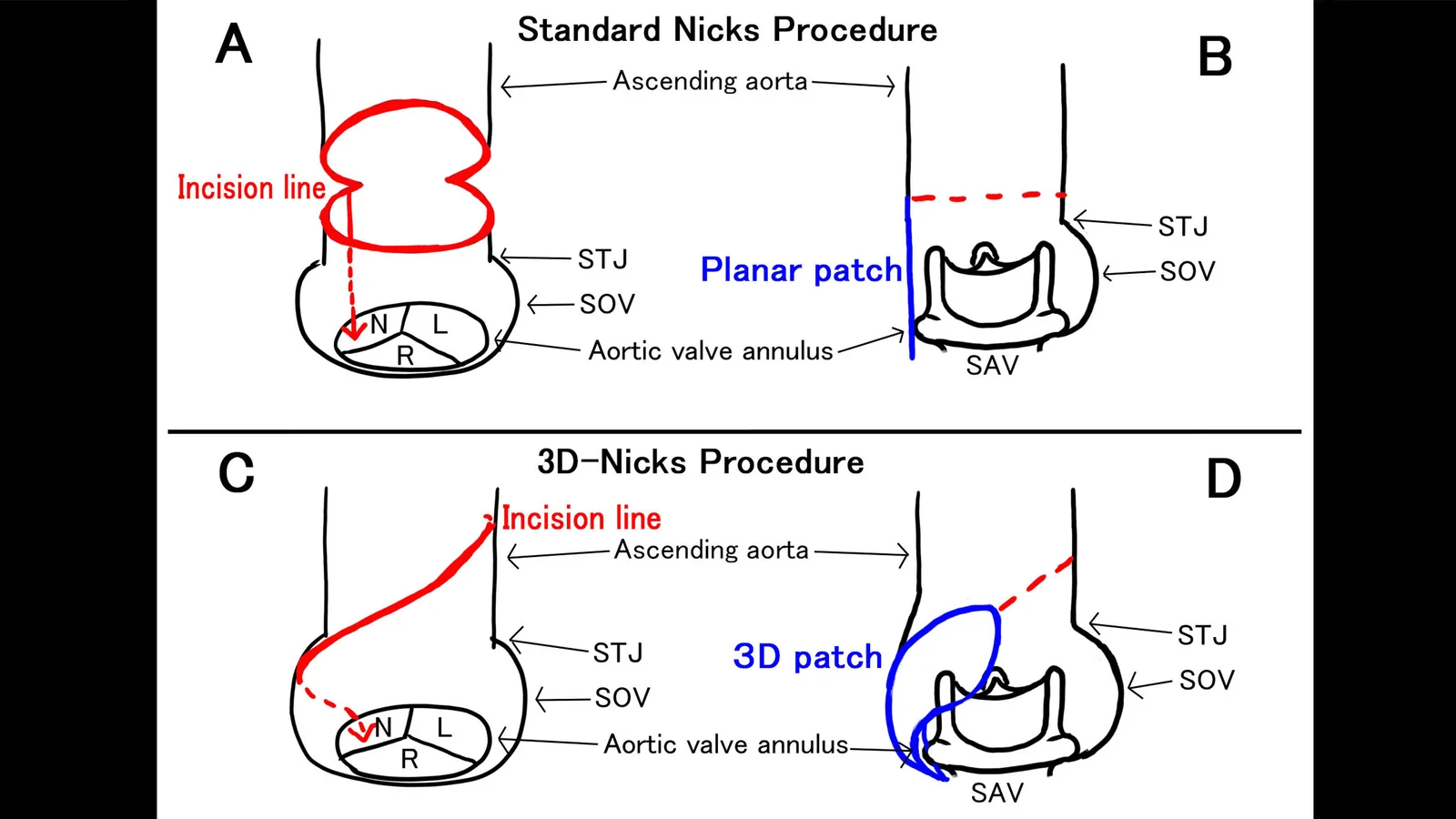
Differences between the standard Nicks procedure and the 3D-Nicks procedure A: Incision line (red line) of the standard Nicks procedure. B: How the planar patch makes contact in the standard Nicks procedure (blue line). C: Incision line (red line) of the 3D-Nicks procedure. D: How the 3D patch makes contact in the 3D-Nicks procedure (blue line). L, left coronary cusp; R, right coronary cusp; N, non-coronary cusp; STJ, sinotubular junction; SOV, sinus of Valsalva; SAV, surgical aortic valve. Image credit: Created by the authors.

On the other hand, in the 3D-Nicks procedure, the three-dimensional patch has the potential to allow for expansion while maintaining its circular shape, resulting in circumferential expansion of the NCC side, including the area opposite the patch, as shown in Figure 4. This ensures the prosthetic valve is positioned neatly in the center. This maintains an appropriate distance between the VTC and VTSTJ, avoiding situations where the stent strut comes into contact with the aortic wall due to distorted base expansion (Figure 6D).

In standard SAVR, the aortotomy is typically approached via a transverse incision above the STJ; the Nicks procedure involves extending this incision longitudinally from the transverse line toward the NCC (Figure 6A). In contrast, we perform the J-shaped incision on the ascending aorta, starting with a longitudinal-oblique cut cranial to the STJ and curving toward the NCC (Figure 6C). This secures a working space for the SAVR procedure, and by applying a three-dimensional patch to this J incision line, the patch will be expanded to include the STJ.

The procedure itself is relatively simple and safe; the three-dimensional patch can be prepared before the initiation of extracorporeal circulation, and the boat-shaped space made by the ellipsoidal patch avoids compression of the stent-post during closure of the aortotomy.

The 3D patch is actually applied diagonally from the lower end of the incision along the J-shaped incision in the aorta, but assuming that it is applied almost vertically to the incision, if the width is designed to be the difference in circumference determined from the target aortic diameter and the preoperative diameter plus about 5 mm of seam allowance, it will be expanded to approximately the target diameter. Also, regarding the height of the patch, the height may be reduced by adding folds to make it three-dimensional, or by having the patch touch diagonally, but it is probably best to design it to approximately the height you want to increase plus the seam allowance (Figure 2B).

In a report simulating the risk of coronary artery obstruction during future TAV-in-SAV procedures based on pre- and post-SAVR contrast-enhanced CT measurements of 115 patients, the mean measurements for high-risk cases (n=33; defined as VTC <4 mm or VTSTJ <2 mm) were as follows: annulus diameter 23.6 mm, prosthetic valve inner diameter 19.0 mm, SOV diameter 28.8 mm, and STJ diameter 24.7 mm [4]. In the present case, although the preoperative SOV diameter (27 mm) and STJ diameter (20 mm) were smaller than the mean values ​​observed in the high-risk group of the aforementioned report, the use of the 3D-Nicks technique resulted in an expansion of the SOV diameter to 30.2-36.8 mm and the STJ diameter to 31.8 mm. Consequently, the VTC exceeded 5 mm, and the VTSTJ exceeded 6 mm, effectively avoiding the risk of coronary artery obstruction; this suggests that root enlargement using the 3D-Nicks technique was effective.

In this case, had the standard Nicks procedure been performed instead of the 3D-Nicks procedure--maintaining an STJ diameter of 20 mm and an SOV height of 13 mm--the VTSTJ would have been 0 or less regardless of the THV used. Furthermore, the STJ would have been positioned below the "risk plane" (the height at which the leaflets are fixed in an open position), suggesting that TAV-in-SAV would have been more difficult due to the risk of sinus sequestration.

There are controversies regarding additional surgery for patients with a small aortic annulus; some reports indicate that aortic annular dilation increases complications and perioperative mortality [19]; on the other hand, there are reports that showed no significant difference in mortality risk, myocardial infarction, stroke, complete atrioventricular block, or re-sternotomy for hemostasis in isolated SAVR cases [20].

Furthermore, in cases where a valve of sufficient size can be implanted without enlarging the aortic annulus and the aortic root is narrow, it appears to be feasible to perform enlargement using a 3D patch on the SOV alone, without incising the annulus. This procedure may offer advantages for patients with small aortic root anatomy who are expected to require reoperation for SVD in the future.

Limitations

As a single case report, these findings demonstrate proof-of-concept and anatomical feasibility rather than definitive clinical superiority or universal safety. Further prospective cohort studies with serial CT imaging are required to evaluate the reproducibility and long-term outcomes of the 3D-Nicks technique across broader patient populations.

## Conclusions

In a patient with a small aortic annulus and root anatomy, SAVR with the 3D-Nicks procedure maintained aortic root dimensions for 14 years and allowed subsequent TAV-in-SAV with a supra-annular valve without coronary obstruction. This suggests 3D-Nicks may be a feasible strategy for patients with a small aortic root who may require future valve-in-valve intervention. Further studies are needed to assess durability and safety.
